# Targeting the epidermal growth factor receptor

**DOI:** 10.1038/sj.bjc.6601921

**Published:** 2004-07-06

**Authors:** B F El-Rayes, P M LoRusso

**Affiliations:** 1 Division of Hematology and Oncology, Karmanos Cancer Institute, Wayne State University, USA

**Keywords:** EGFR, gefitinib, erlotinib, cetuximab

## Abstract

The epidermal growth factor receptor (EGFR) is a member of the erbB family of tyrosine kinase receptors (RTK). The EGFR is involved in cell proliferation, metastasis and angiogenesis, and is expressed in a large proportion of epithelial tumours. The two main classes of EGFR inhibitors in clinical trials are the RTK inhibitors and the monoclonal antibodies. The clinical development of EGFR inhibitors has introduced new challenges to the design of phase I, II, and III trials. Both classes of agents can be safely administered at doses sufficient to inhibit the EGFR system. Receptor tyrosine kinase inhibitors have been extensively evaluated in non-small-cell lung cancer. In this setting, gefitinib has demonstrated activity in patients who fail initial chemotherapy. Monoclonal antibodies have been developed in combination with cytotoxic chemotherapy in several tumour types, most notably colorectal and head and neck cancer. The preliminary results suggest an increase in response rate and time to progression with the combination of cetuximab and chemotherapy in both disease models. Future issues in the development of EGFR inhibitors include the identification of biologic predictors of response, combination with other targeted agents, and their utilisation in earlier stage malignancies.

The epidermal growth factor receptor (EGFR; erbB1) is a member of the tyrosine kinase receptor family, which includes HER2/neu (erbB2), erbB3, and erbB4 (Olayioye *et al*, 2000; Yarden, 2001). The ErbB receptors are present at the cell surface and share a common structure composed of an extracellular ligand-binding domain, transmembrane segment, and an intracellular tyrosine kinase domain (Yarden, 2001). In normal tissue, the ErbB receptors are activated by a variety of receptor-specific ligands. The ligands specific to the EGFR are epidermal growth factor and transforming growth factor-*α* (TGF-*α*) (Yarden, 2001). After ligand binding, the receptors form homo- or heterodimeric complexes activating the tyrosine kinase domain (Olayioye *et al*, 2000; Yarden, 2001). Subsequently, intracellular proteins involved in signalling pathways are phosphorylated and activated, resulting in modulation of gene transcription (Schlessinger, 2000).

The function of the ErbB receptors is dysregulated in several malignant disorders including among others lung, breast, colorectal, squamous cell cancer of the head and neck (SCCHN), and prostrate cancer (Salomon *et al*, 1995; Mendelsohn, 2002). Mechanisms involved in the activation of the ErbB receptors include: (1) receptor overexpression (Hirsch *et al*, 2003), (2) mutant receptors resulting in ligand-independent activation (Hirsch *et al*, 2003; Moscatello *et al*, 1995), (3) autocrine activation by overproduction of ligand (Prenzel *et al*, 1999) or (4) ligand-independent activation through other receptor systems such as the urokinase plasminogen receptor (Liu *et al*, 2002). Activation of the EGFR is involved in malignant transformation and tumour growth through the inhibition of apoptosis, cellular proliferation, promotion of angiogenesis, and metastasis.

At the cellular level, three major signalling pathways mediate the downstream effects of EGFR activation (Figure 1

**Figure 1:**
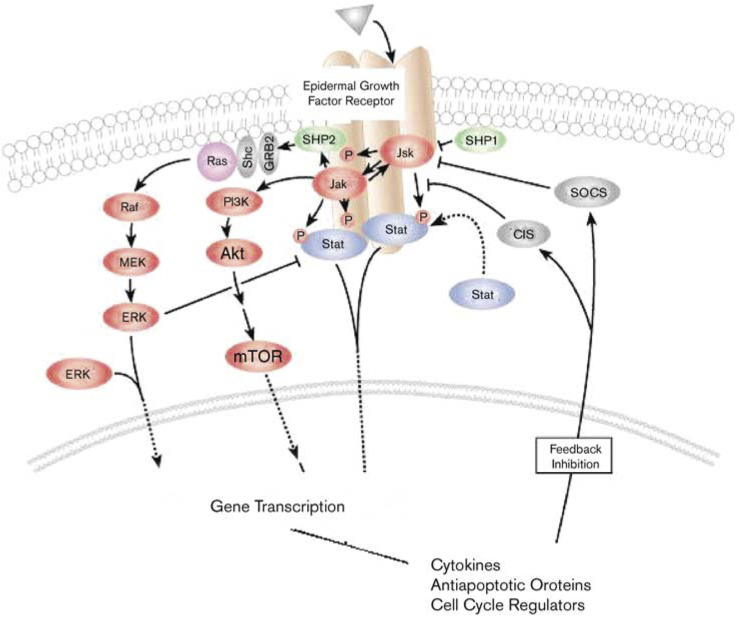
The EGFR signalling pathways. After ligand activation, the EGFR phosphorylates and activates the Ras-Raf-MAP kinase, PI-3K/Akt, and Stat/Jak pathways. This in turn results in activation of transcription factors and modulation of the cell cycle, growth, apoptosis, and angiogenic processes.

). The first pathway involves the Ras-Raf-MAP kinase pathway (Lewis *et al*, 1998). The second pathway involves phosphatidylinositol 3-kinase (PI-3 K) and Akt (Chan *et al*, 1999; Vivanco and Sawyers, 2002). The third pathway involves the stress-activated protein kinase pathway, involving Jak/Stat and protein kinase C (Sato *et al*, 1983; Boudny and Kovarik, 2002).

## STRATEGIES TARGETING THE EGFR PATHWAY

Four strategies for targeting the EGFR are at different stages of development. These include: (1) monoclonal antibodies against the EGFR (Sato *et al*, 1983), (2) inhibition of the receptor tyrosine kinase (RTK) domain (Lichtner *et al*, 2001), (3) inhibition of receptor trafficking to the cell membrane (Yamazaki *et al*, 1998), and (4) inhibition of EGFR synthesis through antisense oligonucleotides (Ciardiello *et al*, 2001b). Only the monoclonal antibody and RTK inhibitor class of agents have been evaluated through phase III trials.

### Monoclonal antibodies

Monoclonal antibodies bind to the extracellular domain of the EGFR and inhibit ligand binding to the receptor (Sato *et al*, 1983). After binding to the EGFR, the monoclonal antibodies induce receptor dimerisation and downregulation. Cetuximab (IMC-C225, Erbitux ImClone Systems Inc, New York, NY, USA), ABX-EGF (Abgenics, San Francisco, CA, USA), and EMD 72000 are monoclonal antibodies directed against the EGFR that are currently in clinical trials. Another class of monoclonal antibodies consists of bispecific antibodies that can bind the EGFR and an immunologic effector cell (Negri *et al*, 1995; Tosi *et al*, 1995; Curnow, 1997). Examples of this class of agents include M26.1, MDX-447, and H22-EGF. These agents have shown promising activity in early clinical trials (Negri *et al*, 1995; Tosi *et al*, 1995; Curnow, 1997).

### Receptor tyrosine kinase inhibitors

Receptor tyrosine kinase inhibitors compete with ATP for the intracellular catalytic site of the EGFR. In contrast to the monoclonal antibodies, this class of agents does not downregulate the EGFR. Receptor tyrosine kinase inhibitors differ with respect to reversibility of inhibition and specificity to the EGFR *vs* the other ErbB receptors. Based on these differences, four different classes of RTK inhibitors can be identified and these include: (1) reversible EGFR inhibitors (e.g. gefitinib, erlonitib), (2) irreversible EGFR inhibitors (e.g. EKB-569), (3) reversible dual-ErbB inhibitors (e.g. GW2016), and (4) irreversible pan-ErbB inhibitors (e.g. CI-1033) (Mendelsohn and Baselga, 2003).

### Comparison of the monoclonal antibody and RTK compounds

Both these classes of agents result in downregulation of the MAPK, PI3K/Akt, and Jak/Stat signal transduction pathways (Bruns *et al*, 2000; Albanell *et al*, 2001). Monoclonal antibodies also downregulate EGFR expression, while RTKs inhibit receptor phosphorylation without affecting expression. At the cellular level, EGFR inhibitors result in cell cycle arrest at the G1 phase (Wu *et al*, 1995; Busse *et al*, 2000), decrease tumour neovascularisation by downregulating expression of angiogenic mediators such as vascular endothelial growth factor (VEGF) (Perrotte *et al*, 1999; Ciardiello *et al*, 2001a), and promote apoptosis (Moyer *et al*, 1997; Liu *et al*, 2000). While monoclonal antibodies require an intact EGFR ligand-binding domain to be active, the RTK inhibitors are active against mutated forms of the EGFR.

At the clinical level, several differences between RTK inhibitors and monoclonal antibodies exist. The RTK compounds are orally administered while the monoclonal antibodies require intravenous administration. While both classes of agents are associated with acenform rash (Baselga *et al*, 2000; 2002), only RTK inhibitors have been associated with gastrointestinal toxicity (Baselga *et al*, 2002; Herbst *et al*, 2002). The preliminary results of clinical trials also suggest different disease-specific activity for each class of agents. For example, cetuximab (Saltz *et al*, 2001a; 2002) and EMD 72000 (Tewes *et al*, 2002) are both active in colorectal cancer, in contrast to erlonitib (Townsley *et al*, 2002) and gefitinib (Seymour *et al*, 2002), which have failed to demonstrate activity against this tumour type, but have shown activity against non-small-cell lung cancer (NSCLC).

## RESULTS OF THE CLINICAL TRIALS EVALUATING THE RTK INHIBITORS

### Gefitinib

In the initial phase I clinical trials, patients were treated with escalating doses of gefitinib (50–925 mg day^−1^) for 14 days of a 28-day cycle (Ranson *et al*, 2002; Nakagawa *et al*, 2003). In these trials, the maximal tolerated dose (MTD) was 700 mg day^−1^. The dose-limiting toxicities were diarrhoea and aceniform rash. Objective responses were observed across all doses starting at the 225 mg day^−1^ dose, raising the possibility that inhibition of the EGFR may be achieved at doses lower than the MTD. In order to determine the optimal biologic dose for gefitinib, two identical multicentre Phase I pharmacodynamic (PD) trials were performed in patients with five tumour types known to express EGFR (NSCLC, SCCHN, ovarian, colorectal, or prostate cancer) (Baselga *et al*, 2002; Herbst *et al*, 2002). Secondary objectives were to determine the pharmacokinetic (PK) profile, to investigate the feasibility and sensitivity of the Functional Assessment of Cancer Therapy (FACT) questionnaire and the seven-item Lung Cancer Subscale (LCS) of FACT in assessing improvements in quality of life and disease-related symptoms, respectively. Dose escalation proceeded until the MTD (800 mg day^−1^) was determined. Common adverse events were mild dose-related skin toxicity and diarrhoea. Biologically relevant plasma concentrations were maintained at doses ⩾150 mg day^−1^, and skin biopsies demonstrated EGFR inhibition at the same dose as well as inhibition of the downstream signalling pathways involving MAPK, p27, and keratinocyte proliferation index (Albanell *et al*, 2002). Both the LCS and FACT questionnaires were found to be feasible and sensitive tools with which to assess improvements in these areas. Patients with NSCLC who had stable disease for ⩾6 months also had improvements or stabilisation in disease-related symptoms (LCS scores), while those patients with disease progression had worsened LCS scores (LoRusso *et al*, 2003). These trials reported the utility of alternative end points in early clinical trials of novel, targeted, anticancer agents.

Based on the phase I trials, two dose levels were selected for Phase II/III studies: 250 and 500 mg day^−1^. The former is above the lowest dose shown to produce biologic and antitumour activity, thereby ensuring adequate gefitinib drug exposure. Pharmacokinetics from phase I trials also identified plasma levels greater than the targeted cell line IC_90_ values (100 ng ml^−1^) in 100% of patients treated at this dose. The 500 mg dose was the highest dose tolerated by most patients on a chronic daily dosing schedule. It also provided greater exposure than the 250 mg dose.

Two large, dose-randomised, double-blind, parallel-group, multicentre Phase II trials (IDEAL 1 and 2, Iressa™ Dose Evaluation in Advanced Lung cancer) independently evaluated the activity of 250 and 500 mg day^−1^ gefitinib in a combined total of 425 patients with advanced NSCLC who failed prior chemotherapy (Fukuoka *et al*, 2003; Kris *et al*, 2003). In both trials, fewer and less severe side effects were observed using 250 mg day^−1^ compared with 500 mg day^−1^, while no differences in efficacy end points (response rate, disease control rate, overall survival, and symptom improvement) were seen between the two doses. Response rates ranged from 9 to 19% and, overall, approximately 40% of patients experienced disease control and symptom improvement. These two trials resulted in the recommendation of the 250 mg dose for use in further clinical trials.

Two randomised trials (INTACT 1 and 2, Iressa NSCLC Trial Assessing Combination Treatment) evaluated the effect of combining gefitinib and chemotherapy as first-line therapy for NSCLC. In the first trial, 1250 patients were randomised to receive gemcitabine and cisplatin with either placebo or gefitinib at either 250 or 500 mg day^−1^ (Giaccone *et al*, 2002). In the second trial, 1037 patients were randomised to receive carboplatin and paclitaxel with either placebo, gefitinib 250 or 500 mg day^−1^ (Herbst *et al*, 2003). In both trials, no difference in survival, progression-free survival or symptom control was observed between the gefitinib/chemotherapy and the chemotherapy alone groups. One possible interpretation for the lack of synergy between gefitinib and cytotoxic agents is related to the G1 arrest of cells continuously exposed to gefitinib. Human cancer xenograft models comparing pulsatile to continuous administration of gefitinib in combination with paclitaxel demonstrated superior tumour kill with the pulsatile schedule (Solit *et al*, 2003). Based on these preclinical data, trials designed to evaluate pulsatile administration of gefitinib in combination with cytotoxic agents in NSCLC are being conducted.

Cohen *et al* (2002) reported the results of gefitinib (500 mg day^−1^) in 52 patients with recurrent SCCHN. Of the 40 response-evaluable patients, eight patients had an objective response and 14 patients had stable disease. Phase II trials of gefitinib in prostate (Moore *et al*, 2002), breast, colorectal (Seymour *et al*, 2002), and gastric cancer have been reported or are ongoing. Table 1

**Table 1:** Summary of clinical trials evaluating RTK inhibitors

**Agent**	**Disease**	**Trial design**	**Results**
Gefitinib	NSCLC		
	IDEAL 1	Randomised phase II trialSecond- and third-line therapySingle-agent gefitinib at 250 and 500 mg day^−1^ doses	Response rate 18%Stable disease 54%No difference between the two arms
	IDEAL 2	Randomised phase II trialThird-line therapy	Response rate 8 8–11%Stable disease 42%
		Single-agent gefitinib at 250 and 500 mg day^−1^ doses	No difference between the two arms
	INTACT 1	Randomised phase III trial Gemcitabine/cisplatin with or without gefitinib	No difference between the three arms. (median survival 11.1, 9.9, and 9.9 months for placebo, 250 mg, 500 mg arms, respectively)
	INTACT 2	Randomised phase III trial Carboplatin/Taxol with or without gefitinib	No difference between the three arms. (median survival 9.9, 9.8, and 8.7 months for placebo, 250 mg, 500 mg arms, respectively)
	Prostate cancer	Phase II trial in hormone refractory disease. Patients randomised to 250 and 500 mg day^−1^ dose	No objective or PSA responses observed
	SCCHN	Phase II single-agent study	Response rate 11%
	Colorectal cancer	Second-line therapy. Gefitinib dose 750 mg day^−1^	No responses
			
Erlonitib	NSCLC	Phase II trial Second/third-line therapy	Overall response rate 12% 1-year survival 40%
	Ovarian cancer	Phase II trial Previously treated patients	Overall response rate 6% Stable disease in 20%
	SCCHN	Phase II trial Previously treated patients with local or metastatic recurrence	Overall response rate 5%
	Hepatocellular cancer	Phase II previously untreated patients	Overall response 50% Median time to progression 3.2 months

summarises the results of these trials.

### Erlonitib

Based on the phase I trial, the MTD of erlonitib is 150 mg day^−1^ (Hidalgo *et al*, 2001). Erlotinib was evaluated in a phase II trial in 57 patients with non-small-cell lung cancer, who had failed first-line chemotherapy (Perez-Soler *et al*, 2001). The overall response rate was 12% and the 1-year survival was 40%. Currently, erlonitib *vs* placebo is being evaluated in a phase III trial in patients with refractory NSCLC and in first-line setting with combination chemotherapy.

Erlonitib has also been evaluated in phase II trials in ovarian (Finkler *et al*, 2001), SCCHN (Senzer *et al*, 2001), and hepatocellular carcinoma (Philip, 2004). A summary of the results and design of the above studies is provided in Table 1.

## RESULTS OF CLINICAL TRIALS EVALUATING MONOCLONAL ANTIBODIES AGAINST EGFR

### Cetuximab

Phase I trials have established the optimal biologic dose range of cetuximab to be 200–400 mg m^−2^ (Baselga *et al*, 2000). At this dose range, cetuximab downregulates EGFR and inhibits downstream signalling. The major toxicity was aceniform rash. Allergic or anaphylactic reactions were observed in 2% of the patients. Cetuximab has been evaluated in colorectal, SCCHN, NSCLC, and pancreatic cancer.

In contrast to the development of RTK inhibitors, early clinical trials with cetuximab have focused on combination therapy with cytotoxic agents. This was based on the nonoverlapping toxicity as well as the experiments in cell culture and human xenograft models demonstrating the potentiation of the effects of cytotoxic agents by cetuximab. Table 2

**Table 2:** Summary of clinical trials evaluating cetuximab

**Disease**	**Cytotoxic agents**	**Trial design**	**Results**
Colorectal cancer	Irinotecan	Phase II trial in patients progressing on irinotecan	Partial response 22.5%Stable disease 7%
		Phase III trial in patients progressing on irinotecan comparing cetuximab to the combination	Response rate (23 *vs* 11%), stable disease (55.5% *vs* 32%), and TTP (4.1 *vs* 1.5 months) significantly improved by the combination
			
SCCHN	Cisplatin	Phase II trial in patients progressing on cisplatin	Response rate 11%
		Phase III trial in patients progressing on cisplatin comparing cetuximab to the combination	Significant improvement in response rate but not in survival
			
Pancreatic cancer	Gemcitabine	Phase II trial in previously untreated patients	Overall response rate 51%. Median TTP 12 weeks
			
NSCLC	Cisplatin/vinorelbine	Randomised trial in the first line setting	Response rate 50% in the cetuximab/chemotherapy arm *vs* 29% in the chemotherapy only arm

summarises the results of recent trials involving monoclonal antibodies against the EGFR. In a phase II trial, 120 patients with colorectal cancer who had progressed on irinotecan were treated with cetuximab and irinotecan. The observed response rate was 22.5% (Saltz *et al*, 2001b). A subsequent phase II trial demonstrated that the response rate to cetuximab in a similar group of patients was 11%, suggesting that cetuximab can modulate the mechanism of irinotecan resistance (Saltz *et al*, 2002). Cunningham *et al* (2003) reported on a phase III trial randomising patients with colorectal cancer, who had progressed on irinotecan to cetuximab with or without irinotecan. A total of 329 patients were enrolled. Response rate (cetuximab/irinotecan 23 *vs* cetuximab 11%, *P*=0.074) and time to progression (cetuximab/irinotecan 4.1 months *vs* cetuximab 1.5 months, *P*<0.001) were significantly improved by the combination. No significant difference in survival was observed. A phase II trial evaluating cetuximab in patients with advanced SCCHN refractory to platinum-based regimens has recently been reported (Baselga *et al*, 2003). In all, 75 patients were enrolled in the study. The observed response rate was 11%. A phase III trial compared cisplatin and cetuximab to cisplatin and placebo in patients with recurrent SCCHN previously treated with cisplatin (Burtness *et al*, 2003). A total of 118 patients were enrolled in the study. The response rates were significantly higher in the group of patients on the combination arm (25.7 *vs* 10.2%, *P*=0.048). There was no significant difference with respect to median progression-free survival and overall survival between the two arms of the study.

Preliminary results of a randomised trial comparing cisplatin/vinorelbine with or without cetuximab in previously untreated patients with NSCLC have been reported (Gatzemeier *et al*, 2003). In contrast to the INTACT trial design, only patients with EGFR expressing tumours were enrolled in the study. Of the 73 patients screened, only 65 patients (89%) expressed the EGFR. A total of 56 patients were enrolled in the study. The overall response rate was higher in the cetuximab arm (50 *vs* 29%). The final results of this study are pending.

A Phase II trial evaluating cetuximab and gemcitabine in advanced chemo-naïve pancreatic cancer was designed (Abbruzzese *et al*, 2001). In all, 41 patients were treated with weekly cetuximab and gemcitabine. The end points were objective response and time to progression. The overall response rate was 51%, with 12% partial response and 39% stable disease. Time to progression (TTP) was 12 weeks, which is longer than the historical control with gemcitabine (median TTP 8 weeks). The Southwestern Oncology Group (SWOG) is currently comparing gemcitabine with and without cetuximab in pancreatic cancer.

## FUTURE DIRECTIONS

### Predictors of response

The advantages of defining predictors of response include: preventing the exposure of patients to potentially harmful and/ or ineffective agents, increasing the effectiveness of therapy through selecting a group of patients with a higher likelihood of response, and identifying patient populations that require different therapies. Since response to other targeted agents such as herceptin and tamoxifen depends mainly on the level of expression of the target, several trials have focused on defining a similar association in the EGFR system. Saltz *et al* (2001a) found no association between EGFR expression by immunohistochemistry in colorectal cancer and response to cetuximab. Similarly, no association was found between response to cetuximab and EGFR expression in SCCHN (Baselga *et al*, 2003), response to gefitinib in NSCLC (Bailey *et al*, 2003), and breast cancer (Iacobuzio-Donahue *et al*, 2003).

The baseline activation of the EGFR and the dependence of the downstream signalling pathways on the EGFR are other potential predictors of response. For example, preclinical models suggest that cells with mutant PTEN phosphatase resulting in EGFR-independent activation of the Akt pathway are resistant to RTK inhibitors (Anido *et al*, 2003). To define the translational worth of these markers, a prospective trial should be designed to incorporate an evaluation of the EGFR and the downstream signaling pathway status pre and post treatment in order to define the predictors of response to EGFR inhibitors. These trials will require serial tumour biopsies, which raise ethical and financial issues related to subjecting patients to invasive procedures. These trials could also help in defining features present in pre-treatment biopsies that could predict for response. An example of such a trial is the recently reported phase I trial of EMD 72000 in patients with colorectal cancer. In this study, only tumours with low baseline phosphorylated Akt that was inhibited post treatment had a response to EMD 72000. These results suggest that the Akt might play a central role in the antitumour effects of EGFR inhibitors. Another approach to identify predictors of response to EGFR blockade is to utilise gene microarrays. The advantage of this design is that it allows investigators to assay the effects of the EGFR inhibitors on the expression of a large number of proteins. Such trial designs would still require serial tumour biopsies.

### Combination therapy involving EGFR inhibitors

As discussed previously, several recent trials have focused on combining EGFR inhibitors with cytotoxic chemotherapy. Other combinations at different stages of development include EGFR inhibitors with other targeted agents, or with radiation therapy. Cancer cells have several dysregulated and redundant pathways; therefore, combining targeted agents may be necessary in order to achieve the desired modulation of a cellular pathway. Combining inhibitors of the EGFR with inhibitors acting on the downstream signalling pathway such as MAPK or Akt could potentially result in an improved inhibition of these pathways translating into increased antitumour effects. These combinations are currently being evaluated in preclinical models. Activation of the EGFR system results in transcription of several proteins such as VEGF and cyclooxygenase-2. Therefore, inhibiting the EGFR can downregulate the expression of these targets, facilitating their inhibition by target-specific agents. The preliminary results of a phase I/II trial evaluating bevacizumab and erlonitib in patients with NSCLC have been recently reported (Mininberg *et al*, 2003). The preliminary results indicate that both agents can be safely administered at full dose. A phase II trial at Wayne State University is evaluating celecoxib and gefitinib in NSCLC. Since RTK inhibitors and monoclonal antibodies inhibit the EGFR system by different mechanisms, their antitumour effects could potentially be improved by combining them. Similarly, since EGFR and ErbB2 can heterodimerise and both receptors are simultaneously overexpressed in several disease models, combining herceptin with an EGFR inhibitor might be necessary to inhibit both receptors. The results of clinical trials exploring such combinations have not yet been reported. Cetuximab was safely combined with radiation therapy in a phase II trial of SCCHN (Robert *et al*, 2001). In all, 13 complete and two partial responses were observed in the 16 patients enrolled in the study. Encouraged by these results, a phase III trial of radiation with or without cetuximab is ongoing.

### Role of EGFR inhibitors in early-stage disease

Epidermal growth factor receptor inhibitors have demonstrated significant activity in patients with metastatic NSCLC, who have failed cytotoxic chemotherapy. These results raise the possibility of a role for EGFR inhibitors in locally advanced NSCLC. Currently, SWOG is conducting a randomised trial in patients with stage III NSCLC. Patients enrolled in this study will receive definitive chemo-radiotherapy, followed by docetaxel with subsequent randomisation to either gefitinib or placebo. The low incidence of toxicity associated with the EGFR inhibitors has also raised the possibility of a potential role for these agents in the adjuvant setting. SWOG is currently conducting a phase III trial randomising patients with stage I and II NSCLC to either gefitinib or placebo after resection. The results of these trials will help define the role of targeted agents after definitive treatment of early-stage and locally advanced NSCLC.

## CONCLUSION

The EGFR inhibitors have already demonstrated activity in several advanced stage cancers including NSCLC, colorectal, and squamous cell carcinomas of the SCCHN. The role of EGFR inhibitors in early-stage disease is currently being evaluated. The preclinical and clinical development of this class of agents has required novel trial designs that could be incorporated into future trials involving other novel targeted therapies.
